# Genome-wide association study of hippocampal atrophy rate in non-demented elders

**DOI:** 10.18632/aging.102470

**Published:** 2019-11-23

**Authors:** Yu Guo, Wei Xu, Jie-Qiong Li, Ya-Nan Ou, Xue-Ning Shen, Yu-Yuan Huang, Qiang Dong, Lan Tan, Jin-Tai Yu

**Affiliations:** 1 Department of Neurology, Qingdao Municipal Hospital Affiliated to Qingdao University, Qingdao, China; 2 Department of Neurology and Institute of Neurology, Huashan Hospital, Shanghai Medical College, Fudan University, Shanghai, China

**Keywords:** hippocampal atrophy rate, cognitive decline, genome-wide association study

## Abstract

Hippocampal atrophy rate has been correlated with cognitive decline and its genetic modifiers are still unclear. Here we firstly performed a genome-wide association study (GWAS) to identify genetic loci that regulate hippocampal atrophy rate. Six hundred and two non-Hispanic Caucasian elders without dementia were included from the Alzheimer’s Disease Neuroimaging Initiative cohort. Three single nucleotide polymorphisms (SNPs) (rs4420638, rs56131196, rs157582) in the *TOMM40*-*APOC1* region were associated with hippocampal atrophy rate at genome-wide significance and 3 additional SNPs (in *TOMM40* and near *MIR302F* gene) reached a suggestive level of significance. Strong linkage disequilibrium between rs4420638 and rs56131196 was found. The minor allele of rs4420638 (G) and the minor allele of rs157582 (T) showed associations with lower Mini-mental State Examination score, higher Alzheimer Disease Assessment Scale-cognitive subscale 11 score and smaller entorhinal volume using both baseline and longitudinal measurements, as well as with accelerated cognitive decline. Moreover, rs56131196 (P = 1.96 × 10^-454^) and rs157582 (P = 9.70 × 10^-434^) were risk loci for Alzheimer’s disease. Collectively, rs4420638, rs56131196 and rs157582 were found to be associated with hippocampal atrophy rate. Besides, they were also identified as genetic loci for cognitive decline.

## INTRODUCTION

The hippocampus is a vulnerable and plastic structure buried deep in the medial temporal lobe of human body [1]. The atrophic hippocampus is often accompanied by poor memory performance, and changes in the hippocampus provide a neural substrate for cognitive impairment that may be associated with normal aging, post-traumatic stress disorder, recurrent depression, and Cushing's syndrome [2]. Hippocampal atrophy rate has been demonstrated to be closely correlated with cognitive disorders, including the most commonly reported Alzheimer’s disease (AD) and some non-AD disorders, such as frontotemporal dementia (FTD) and impaired memory [3–8]. As for mechanism, hippocampal atrophy rate may relate to the deposition of amyloid-beta (Aβ) and tau [9, 10]. Clinically, the atrophy rate was greater in subjects with normal cognition (NC) who converted to mild cognitive impairment (MCI) or AD than in those who remained stable; it was greater in MCI subjects who converted to AD than in those who remained stable; and it was greater in fast AD progressors than slow ones [11, 12]. Consequently, the reduction in hippocampal volumes over time may be promising in predicting individuals at high risk of developing cognitive decline, monitoring disease trajectories at early stage, and assessing treatment efficacy in clinical practice or drug trials.

Previous genome-wide screening identified novel susceptibility genes for AD using baseline hippocampal volumes as quantitative traits [13]. However, the genetic predictors of longitudinal changes in hippocampal volumes remain poorly understood. Use of quantitative traits in genome-wide association studies (GWAS) provides novel and important insights into broader trends in correlations between genes and their associated pathways [14]. Furthermore, magnetic resonance imaging (MRI) has great advantages in visualizing structural and functional brain changes, such as sufficient sensitivity, non-invasiveness, ease of availability, and good tolerance [15]. And hippocampal volumes can be reliably measured in vivo. Therefore, we conducted a GWAS with longitudinal MRI measures of hippocampal volumes to identify genetic risk factors influencing hippocampal atrophy rate in non-demented elders. These genetic contributors may be involved in cognition-related pathophysiological process.

## RESULTS

### Characteristics of included subjects

This study included 226 NC (111 women, 74.7±5.3 years) and 376 MCI (152 women, 72.3±7.2 years) subjects from the Alzheimer’s Disease Neuroimaging Initiative (ADNI) cohort of non-Hispanic Caucasian ancestry. The summarized characteristics of included subjects were shown in Table 1. MCI group (47.5%) had a higher frequency of ε4 allele within *APOE* gene than NC group (25.7%). MCI group also had bigger baseline whole brain volume (P = 0.020) and smaller baseline hippocampal volume (P < 0.001) compared to NC group (P < 0.001).

**Table 1:** Demographic information.

**Baseline diagnosis**	**NC**	**MCI**	**Total**
Sample size, n (%)	226 (37.5)	376 (62.5)	602
Age at baseline, mean (SD)	74.7 (5.3)	73.2 (7.2)	73.2 (6.7)
Females, n (%)	111 (49.1)	152 (40.3)	263 (43.6)
Education years, mean (SD)	16.4 (2.7)	15.9 (2.9)	16.1 (2.8)
*APOE* ε4 carrier, n (%)	58 (25.7)	178 (47.5)	236 (39.7)
MMSE at baseline, mean (SD)	29.1 (1.1)	27.9 (1.6)	28.4 (1.6)
WBV at baseline, mean (SD)	1,033,459.2 (100,326.4)	1,053,883.0 (108,862.1)	1,046,258.1 (106,216.4)
HPV at baseline, mean (SD) ^a^	7,296.7 (881.2)	6,950.0 (1,124.4)	7,080.1 (1,053.3)

Abbreviations: *APOE, apolipoprotein E*; HPV, hippocampal volume; MCI, mild cognitive impairment; MMSE, Mini-mental State Examination; NC, normal cognition; SD, standard deviation; WBV, whole brain volume.

^a^The MCI group had lower baseline hippocampal volumes compared to the NC group (P < 0.001).

### Single nucleotide polymorphisms (SNPs) associated with hippocampal atrophy rate

**Figure 1:**
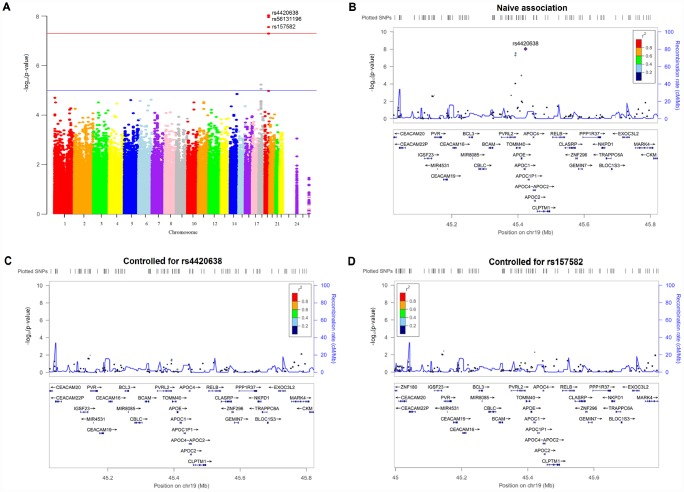
**Manhattan and regional plots for associations with hippocampal atrophy rate.** (**A**) Genome-wide signal intensity (Manhattan) plots showing the －log_10_ (p value) for individual single nucleotide polymorphisms. (**B**) Regional association results for the 45.0 Mb to 45.8 Mb region of chromosome 19. (**C**) Association results for the 45.0 Mb to 45.8 Mb region of chromosome 19 controlling for rs4420638. (**D**) Association results for the 45.0 Mb to 45.8 Mb region of chromosome 19 controlling for rs157582.

**Figure 2:**
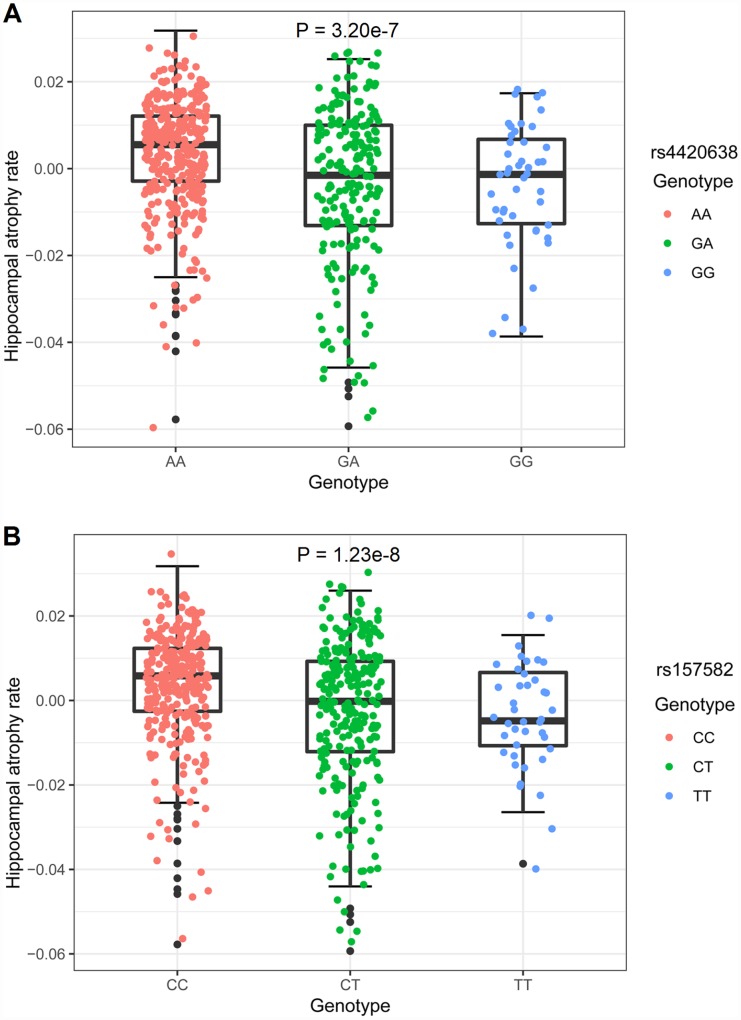
**Hippocampal atrophy rates of different genotypes.** The y-axis showed the hippocampal atrophy rate and the x-axis corresponded to different genotypes. The effect of genotypes on hippocampal atrophy rate was examined with a multiple linear regression model using age, gender and diagnosis as covariates. (**A**) The minor allele of rs4420638 (G) showed association with hippocampal atrophy rate in a dose-dependent manner (P = 3.20 × 10^-7^). (**B**) The minor allele of rs157582 (T) showed association with hippocampal atrophy rate in a dose-dependent manner (P = 1.23 × 10^-8^).

There were 602 individuals identified for GWAS. After adjusting for age, gender, years of education, intracranial volume (ICV), MRI and the first three multidimensional scaling (MDS) components, 3 SNPs on chromosome 19, including rs4420638 in the *APOC1* gene (minor allele frequencies (MAF) = 0.1510, P = 9.32 × 10^-9^), rs56131196 in the *APOC1* gene (MAF = 0.1508, P = 1.10 × 10^-8^) and rs157582 in the *TOMM40* gene (MAF = 0.2937, P = 2.78 × 10^-8^), exhibited genome-wide significant associations with hippocampal atrophy rate (Figure 1A and Table 2). Their association signals disappeared after including *APOE* ε4 dosage as a covariate (Supplementary Figure 2). Strong linkage disequilibrium (LD) (R^2^ = 0.987, D’ = 0.993) between rs4420638 and rs56131196 was found, which was calculated using the 1,000 Genomes European cohort (Supplementary Figure 3). Accordingly, rs4420638 and rs157582 were chosen as the two top SNPs, for which the minor allele of rs4420638 (G) (AA: 0.003478 ± 0.012252, AG: -0.003415 ± 0.017455, GG: -0.003895 ± 0.013808; P = 3.20 × 10^-7^) and the minor allele of rs157582 (T) (CC: 0.003567 ± 0.012731; CT: -0.002717 ± 0.016733, TT: -0.004347 ± 0.012037; P = 1.23 × 10^-8^) were significantly associated with higher hippocampal atrophy rate in a dose-dependent manner (Figure 2).

**Table 2:** Top SNPs associated with hippocampal atrophy rate.

**SNP**	**CHR**	**BP**	**MAF**	**Closest Gene**	**SNP Type**	**BETA**	**P**
rs4420638	19	45422946	G=0.1510	*APOC1*	intron	-0.005454	9.32E-09
rs56131196	19	45422846	A=0.1508	*APOC1*	intron	-0.005557	1.10E-08
rs157582	19	45396219	T=0.2937	*TOMM40*	intron	-0.005351	2.78E-08

Abbreviations: BP, base pair (variant position); CHR, chromosome; MAF, minor allele frequency; SNP, single nucleotide polymorphism.

SNPs mapped closely to the region of top SNPs were also analyzed (Figure 1B). After controlling for the genotypes of the two top SNPs (rs4420638, rs157582) (Figure 1C and 1D), no SNPs showed strong associations with hippocampal atrophy rate in this region, indicating that these nearby SNPs might be driven by the two top SNPs. The Quantile-Quantile plot didn’t show evidence of spurious inflation in test statistics (the genomic inflation factor = 1) due to population stratification or other confounders (Supplementary Figure 4).

Three SNPs were associated with hippocampal atrophy rate at suggestive levels of significance (P < 1 × 10^-5^) (Figure 1A and Supplementary Table 1). These loci comprised SNPs within the *TOMM40* gene (rs2075650) as well as SNPs near the *MIR302F* gene (rs4271662 and rs2900721). The association signals in these SNPs disappeared when *APOE* ε4 dosage was included as a covariate (Supplementary Figure 2 and Supplementary Table 2).

### Impact of rs4420638 and rs157582 on cognitive scores and brain structures

**Figure 3:**
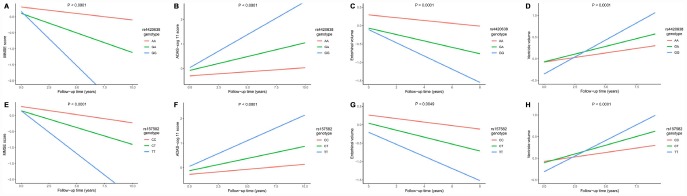
**Impact of rs4420638 and rs157582 on longitudinal measurements in cognitive scores and brain structures.** Associations of rs4420638 with longitudinal measurements in Mini-mental State Examination (MMSE) score (**A**), Alzheimer Disease Assessment Scale-cognitive subscale 11 (ADAS-cog 11) score (**B**), entorhinal volume (**C**) and ventricular volume (**D**) over time. Associations of rs157582 with longitudinal measurements in MMSE score (**E**), ADAS-cog 11 score (**F**), entorhinal volume (**G**) and ventricular volume (H) over time.

Associations of two top SNPs with cognitive scores and brain structures were analyzed. In the cross-sectional analyses, both the minor allele of rs4420638 (G) and the minor allele of rs157582 (T) were associated with lower Mini-mental State Examination (MMSE) score, higher Alzheimer Disease Assessment Scale-cognitive subscale 11 (ADAS-cog 11) score and smaller entorhinal volume (Supplementary Figure 5). In the longitudinal study, subjects with a minor allele of rs4420638 (G) showed faster cognitive decline in MMSE score (P < 0.0001) and ADAS-cog 11 score (P < 0.0001), as well as greater rates of entorhinal atrophy (P = 0.0001) and ventricular enlargement (P < 0.0001). Besides, subjects with a minor allele of rs157582 (T) also showed faster cognitive decline in MMSE score (P < 0.0001) and ADAS-cog 11 score (P < 0.0001), as well as greater rates of entorhinal atrophy (P = 0.0049) and ventricular enlargement (P < 0.0001) (Figure 3).

### Effect of SNPs on risk of cognitive decline

**Figure 4:**
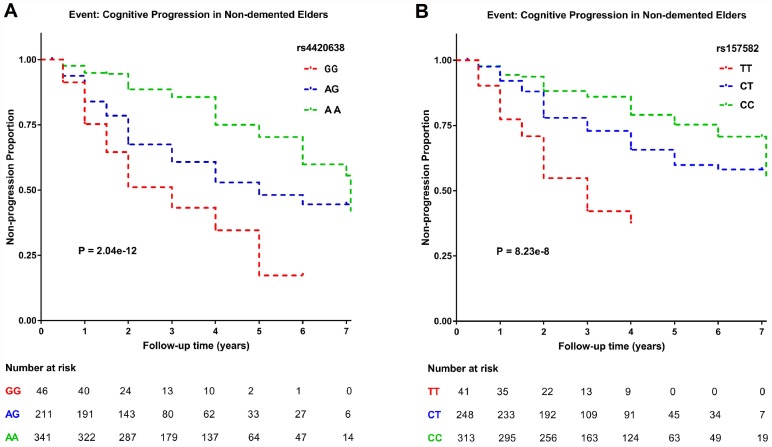
**Kaplan–Meier survival curves for probability of cognitive progression.** Numbers of individuals at risk at different follow-up time points were presented. Survival time was calculated according to the interval from the initial baseline evaluation to cognitive progression. (**A**) Subjects with the minor allele of rs4420638 (G) showed a greater rate of cognitive decline. (**B**) Subjects with the minor allele of rs157582 (T) showed a greater rate of cognitive decline.

After the GWAS, we conducted survival analysis to further explore the influence of two top SNPs on cognitive decline (Figure 4 and Supplementary Table 3). The COX regression analysis was performed on minor allele homozygotes, heterozygotes and major allele homozygotes by including age and gender as covariates. Both the minor allele of rs4420638 (G) (P = 2.04 × 10^-12^) and the minor allele of rs157582 (T) (P = 8.23 × 10^-8^) appeared to accelerate cognitive decline, conferring increased risk to homozygous and heterozygous carriers of the minor allele and confirming the positive direction of effects detected in our GWAS. The GG (hazard ratio (HR) = 3.917, 95% confidence interval (CI) = 2.478 to 6.191) and AG (HR = 2.212, 95% CI = 1.615 to 3.029) genotypes of rs4420638 showed a greater risk of cognitive decline than the AA genotype. Also, the TT (HR = 4.190, 95% CI = 2.502 to 7.016) and CT (HR = 1.704, 95% CI = 1.201 to 2.418) genotypes of rs157582 showed a greater risk of cognitive decline than the CC genotype.

### Bioinformatics analyses

The International Genomics of Alzheimer’s Disease Project (IGAP) is by far the largest genetic epidemiological survey of AD risk, which was performed in two stages comprising a discovery step (stage 1) and a replication step (stage 2). Based on several grand-scale meta-analyses, the IGAP has identified many susceptibility loci for AD [16, 17]. After checking the top loci linked to hippocampal atrophy rate in the stage 1 meta-analysis from IGAP database, we identified rs56131196 (P = 1.96 × 10^-454^) and rs157582 (P = 9.70 × 10^-434^) as risk loci for AD.

As for expression quantitative trait loci (eQTL) analyses, the minor allele of rs4420638 (G) or the minor allele of rs56131196 (A) was upregulated in frontal cortex (P = 0.02) (Supplementary Figure 6). In accordance with the Allen Institute Human Brain Atlas, *APOC1* appeared to be selectively expressed in the hippocampus region. *APOC1* could also be regulated in the temporal and visual cortices (http://human.brain-map.org/). Besides, rs157582 has a modest cis-eQTL effect on *GEMIN7* gene (Z = 3.03, P = 2.41 × 10^-3^) in whole blood (Supplementary Figure 6) [18].

## DISCUSSION

This study was the first to conduct a GWAS of hippocampal atrophy rate in non-demented elders. We identified genome-wide significant associations of 3 SNPs (rs4420638, rs56131196, rs157582) in the *TOMM40*-*APOC1* region with hippocampal atrophy rate and 3 additional suggestive association loci (in *TOMM40* gene and near *MIR302F* gene). The minor allele of rs4420638 (G) and the minor allele of rs157582 (T) showed associations with lower MMSE score, higher ADAS-cog 11 score and smaller entorhinal volume using both baseline and longitudinal measurements, as well as with accelerated cognitive decline. Our findings provide evidence that *TOMM40* and *APOC1* as candidate genes may promote the application of hippocampal atrophy rate as an early biomarker for predicting cognitive progression and detecting disease trajectories.

Both *APOC1* and *TOMM40* are genes adjacent to *APOE*, which showed no significant associations with hippocampal atrophy rate after adjusting for *APOE* ε4 dosage. Although the most concise explanation may be that *APOE* ε4 was driving the observed associations, the extensive LD structure around *APOE* and various associations reported in *APOE* region made it difficult to further explain the result. As in any SNP-trait association, only statistical association was not enough to infer causality [19]. Besides, it’s impossible for us to analyze all potentially causal variants in the region. A previous study also supported that the correlation between *APOE* region and cognitive function was not mediated solely by the *APOE* ε4 allele [20]. And the polymorphic poly-T variant in the *TOMM40* gene among different populations provided more possibilities for the interpretation of our results [21]. Compared with those having shorter poly-T repeats, *APOE* ε3 carriers having a long poly-T repeat in the *TOMM40* gene not only have an earlier age of onset of late-onset AD, but also have decreased memory abilities and grey matter volumes [20]. It seems that the polymorphic poly-T variant in the *TOMM40* gene provides greatly improved accuracy in the estimation of cognitive disorders for *APOE* ε3 carriers. Furthermore, a large-scale longitudinal study reported that both *APOE* ε4 and poly-T repeats in the *TOMM40* gene were associated with cognitive decline, but there was no interaction between the two genes [22]. Thus, it’s difficult to say whether the correlations between *TOMM40*-*APOE*-*APOC1* region and cognitive function were mediated by the *APOE* ε4 allele or by poly-T repeats, or were mediated by more complex mechanisms. More research is warranted to explore the related pathogenesis.

Rs56131196 and rs157582 have been reported in GWAS studies on AD and aging-related verbal memory respectively [23, 24], thus lending validation and confidence to our analytic procedure and results. Previous studies suggested that the elderly carrying *APOC1* gene tended to perform worse in cognitive scores and showed more severe hippocampal abnormalities [25, 26]. Besides, the *APOC1*-knock-out mice might have worse memory performance than those carrying *APOC1* [27]. As for mechanism, ApoC1 (apolipoprotein C1) encoded by *APOC1* is a member of the apolipoprotein family, which may be involved in multiple biological processes comprising cholesterol metabolism and neuronal apoptosis [28]. But the specific mechanisms by which *APOC1* gene modulates the risk of cognitive impairment remain controversial, although some research has been done in this field [26, 29, 30]. The protein that *TOMM40* encodes, TOM40 (translocase of the outer mitochondrial membrane 40), constitutes an external mitochondrial membrane channel that promotes the transport of many aggregating proteins to mitochondria [31, 32]. TOM40 protein may also act as a molecular chaperone that could accelerate the movement of ribosomal pre-proteins through the channel and assemble them in the mitochondria after translation [31, 32]. By mediating the dynamic functions of mitochondria, *TOMM40* may contribute to changes in cognitive status.

Both *TOMM40* and *APOC1* are in strong LD with *APOE* on chromosome 19. Several SNPs in *TOMM40*-*APOE*-*APOC1* region have been detected to be associated with cognitive impairment [19, 33]. Each of these 3 adjacent genes could encode proteins with biological values that may affect cognitive function. However, controversy still exists as to whether the associations between these 3 adjacent genes and cognition are independent of *APOE* allele or are driven by LD with *APOE*. The biochemical interaction between *APOC1* and *APOE* may be associated with cognitive impairment, since the binding of triglyceride lipoproteins to the very low density lipoprotein receptor, mediated by *APOE* allele, could be interfered by over-expressed ApoC1 [26]. Furthermore, *APOC1* could increase the risk of cognitive impairment by modulating lipid metabolism. Additionally, apolipoprotein E (ApoE), amyloid, and synuclein proteins are interactive with Tom40 [34]. The Tom40 protein forms the channel through which amyloid beta protein precursor (APP) and Aβ travel and aggregate to cause mitochondrial abnormalities [32]. There was also evidence for the effects of *APOE* receptors on APP trafficking and Aβ production, and the effects of *APOE* on Aβ aggregation and clearance [35]. Thus, it has been postulated that *APOE* and *TOMM40* genes might share similar mechanisms in mediating disease risk [32].

A functional analysis targeting *TOMM40*-*APOE*-*APOC1* region reported that various *APOE* locus cis-regulatory elements affect both *APOE* and *TOMM40* promoter activity [36]. This indicates that gene expression patterns in this region may be modulated by a complicated transcriptional regulatory structure. Evidence also supported the role of epigenetic mechanisms such as deoxyribonucleic acid (DNA) methylation in the regulation of gene expression [19]. The increase of DNA methylation often leads to down-regulated gene expression by either blocking access of transcriptional factors or enrolling methyl-cytosine-guanine-binding proteins [37]. The repressed gene expression within the region was demonstrated to be correlated with cognitive dysfunction both in blood samples and brain tissues [38–40]. Both the methylation-gene expression and gene expression-cognition associations in the *TOMM40*-*APOE*-*APOC1* region are worth investigating in the future.

There was a suggestive finding in the *MIR302F* gene, which was a member of microRNA (micro ribonucleic acid) family that participated in post-transcriptional regulation of gene expression in multicellular organisms via influencing both the stability and translation of messenger ribonucleic acids [41]. Studies have reported the associations of breast cancer [42], gastric cancer [42, 43] and acute heart failure [44] with *MIR302F* gene, whereas little was known about the mechanism by which *MIR302F* gene correlated with cognitive deterioration. Further investigations are especially warranted to explore how *MIR302F* gene influences the progression of cognitive disorders.

### Limitations

Some limitations must also be acknowledged. Firstly, the sample size was relatively small, which may not be representative of the general population. Secondly, our participants were restricted to non-Hispanic Caucasians and we didn’t explore the diversity among different populations. Thirdly, we applied Bonferroni correction for multiple comparisons and set the MAF threshold at > 0.10, which could enhance statistical power to avoid false-positive results but may miss less common SNPs.

## METHODS

### ADNI dataset

The ADNI was launched in 2003 as a public-private partnership, led by Principal Investigator Michael W. Weiner, MD, VA Medical Center and University of California–San Francisco. The primary goal of ADNI has been to investigate the effectiveness of integrating neuroimaging, genetic/biological markers, as well as clinical and neuropsychological assessments in measuring the progression of MCI and early AD. All ADNI individuals were recruited from over 50 sites across the United States and Canada, and most people were non-Hispanic Caucasians.

### Participants

In this study, 602 non-Hispanic Caucasian non-demented individuals (NC = 226, MCI = 376) were enrolled from the ADNI cohort after applying quality control (QC) procedures. All participants received baseline and periodic clinical and neuropsychological assessments as well as serial MRI. Data used in the preparation for this article were derived from the ADNI database (http://adni.loni.usc.edu/).

A total of 698 samples before QC were available with both GWAS data and hippocampus data. To reduce confounding effects by genetic ancestry that could lead to population stratification, the analysis data was restricted to non-Hispanic Caucasian participants (n = 648). To avoid the impact of AD pathology on results, all participants were restricted to cognitively normal individuals or those with mild cognitive impairment (n = 610). To determine cryptically related individuals and/or sample mix-ups, identify-by-descent estimates and MDS components were conducted using PLINK [45]. This step excluded 5 participants who showed cryptically associated and clustering separately from the other subjects (Supplementary Figure 1), remaining 605 valid samples. Finally, all samples presented tight clustering with the population of European descent using the HapMap cohort.

Individuals were followed up to detect progressive cognitive decline defined as (1) losing > 3 points between the first and last MMSE measurements, (2) developing from NC to MCI or from MCI to dementia, or (3) having a score < 24 at last MMSE [46].

### Hippocampal atrophy rate and QC

Longitudinal hippocampal volume measurements by MRI could be downloaded from the ADNI database, which was conducted by N. Schuff and his colleagues at UCSF via FreeSurfer version 4.3 [47]. The hippocampal atrophy rates were obtained from a mixed-effects model using “arm, lme4 and lmerTest” packages in R software, after controlling for age of entry, gender, number of *APOE* ε4 allele, years of education, baseline diagnosis and total ICV. Individualized rate was then used as a quantitative outcome phenotype for the GWAS. QC was conducted to mitigate the impact of extreme values on statistical results. The mean and standard deviation (SD) of the hippocampal atrophy rate were calculated by experienced operators blinded to clinical data, and the figures greater or smaller than 4-fold SD from the mean value were considered as extreme outliers and removed from this analysis. After eliminating 3 outliers, there were 602 valid subjects left.

### Genotyping and QC

Genotyping for all samples was analyzed by the Illumina Human Hap610-Quad BeadChips featuring 2,379,855 SNPs. QC procedures were implemented with the PLINK software following the stringent criteria: call rates for SNPs > 98%, call rates for individuals > 95%, MAF > 0.10 and Hardy-Weinberg equilibrium test P > 0.001. We restricted the MAF value > 0.10 for SNPs to avoid potentially false-positive results and improve statistical power. Finally, all 602 subjects remained in the analysis and 695,203 SNPs passed QC protocols. The overall genotyping rate in remaining individuals was 99.7%.

### Statistical analyses

One-way analysis of variance (ANOVA) and Turkey’s multiple comparison tests were used to determine the difference in baseline hippocampal volumes among different diagnostic groups. The associations of hippocampal atrophy rate with genetic polymorphisms were determined using multiple linear regression under an additive genetic model in PLINK v1.9 software. To correct for confounding due to population stratification, the first three MDS components were calculated in PLINK and applied as covariates in the regression model. Age of entry, gender, years of education, ICV and MRI were also included as covariates. To investigate the effect of *APOE* ε4 on hippocampal atrophy rate, the above GWAS analyses were repeated with *APOE* ε4 dosage fitted as a covariate. To account for multiple comparisons, Bonferroni correction was applied and thresholds of P < 5 × 10^-8^ and P < 1 × 10^-5^ were used for genome-wide significant and suggestive associations, respectively [48]. Genome-wide associations were visualized using R package “qqman” and regional association plots were generated with the LocusZoom web tool (http://locuszoom.org/). LD analysis was performed using HaploReg v4.1 based on data from the 1000 Genomes Project (EUR). The difference in hippocampal atrophy rates among different genotype groups was determined using a multiple linear regression model in R software after adjusting for age, gender and diagnosis.

R software was also applied to explore the correlations of top SNPs with cognitive scores (MMSE score and ADAS-cog 11 score) and specific brain structures (entorhinal volume and ventricular volume) after adjusting for age, gender, years of education, ICV and MRI. The associations of top SNPs with the above indexes from both cross-sectional and longitudinal perspectives were determined using the multiple linear regression and linear mixed models, respectively. In the survival analysis, Kaplan-Meier survival curves were used to present trajectories of cognitive progression and COX regression model with age and gender as covariates was used to investigate the influence of two top SNPs on cognitive decline.

### Bioinformatics analyses

SNP annotations were performed using the NCBI Database (http://www.ncbi.nlm.nih.gov/SNP/). The IGAP was searched to determine the associations of top SNPs with AD risk. EQTL analyses were conducted using multiple publicly available datasets in human brain tissues (http://BRAINEAC.org; Allen Institute Human Brain Atlas; http://human.brain-map.org/) and the whole blood (http://www.genenetwork.nl/bloodeqtlbrowser/).

## CONCLUSIONS

In summary, we detected 3 genome-wide significant SNPs (rs4420638, rs56131196, rs157582) in the *TOMM40-APOC1* region and 3 suggestive loci (in *TOMM40* and near *MIR302F*) associated with hippocampal atrophy rate among non-demented elders. Since the 3 top SNPs were also identified as genetic loci for cognitive decline, our study indicated that hippocampal atrophy rate may be promising for monitoring cognitive progression.

## Supplementary Material

Supplementary Figures

Supplementary Tables
