# Supplementary material for: Genome-wide association study of hippocampal atrophy rate in non-demented elders
Source: Aging (Albany NY). 2019 Nov 23;11(22):10468–84. doi: 10.18632/aging.102470 (PMC6914394; doi:10.18632/aging.102470)
Supplement: Supplementary Figures [file aging-11-102470-s002.pdf]

SUPPLEMENTARY FIGURES

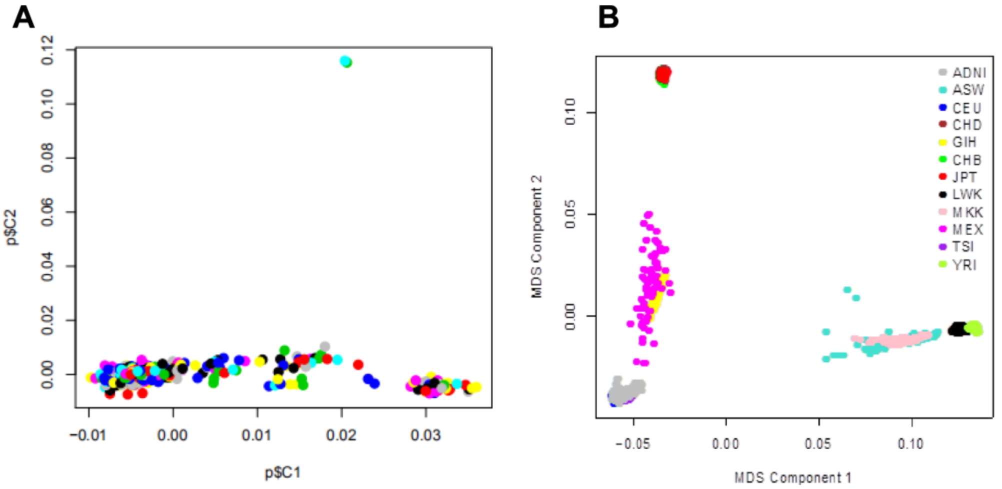

Supplementary Figure 1. Multidimensional scaling plot of ADNI samples. Note: Cryptic relatedness and population substructure were checked with genomic identity-by-descent (IBD) and multidimensional scaling (MDS) components. (**A**) MDS plot of ADNI non-Hispanic Caucasian samples. Samples seemed to form loose clusters and two samples were outliers based on the second MDS component (at top of plot; 031_S_4032 and 031_S_4203), suggesting potential population substructure. To check for cryptic relatedness, which can confound GWAS (genome-wide association study), pairwise identity-by-descent fraction (π) between each pair of samples were calculated using PLINK. Four related sample pairs (π > 0.05) were identified (031_S_4032 and 031_S_4203; 137_S_4466 and 021_S_0159; 023_S_0058 and 023_S_4035; 024_S_2239 and 024_S_4084), which are probably first-degree relatives. No other cryptic relations were identified from the sample, at a threshold of π > 0.05. (**B**) MDS plot of ADNI samples overlaid on HapMap samples. The ancestry of the HapMap participants is shown by the point color. No outlying point was shown. Abbreviations: ADNI, Alzheimer's Disease Neuroimaging Initiative; ASW, African ancestry in Southwest USA; CEU, Utah residents with Northern and Western European ancestry from the CEPH collection; CHB, Han Chinese individuals from Beijing, China; CHD, Chinese in Metropolitan Denver, Colorado; GIH, Gujarati Indians in Houston, Texas; JPT, Tokyo, Japan; LWK, Luhya in Webuye, Kenya; MEX, Mexican ancestry in Los Angeles, California; MKK, Maasai in Kinyawa, Kenya; TSI, Tuscans in Italy; YRI, Yoruba in Ibadan, Nigeria.

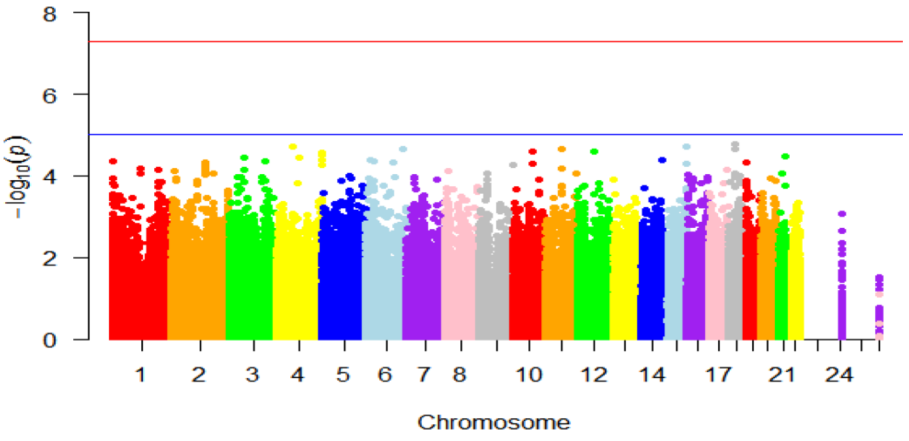

Supplementary Figure 2: Manhattan plot for associations with hippocampal atrophy rates after controlling for *APOE* ε4. Note: Observed $-\log_{10}$ P-values (y-axis) are shown for all tested single nucleotide polymorphisms on each autosomal chromosome (x-axis) after controlling for age, gender, *APOE* ε4 status, education, MRI (magnetic resonance imaging), ICV (intracranial volume) and the first three principal components. No genome-wide significant associations ($P < 5 \times 10^{-8}$; red line) and suggestive associations ($P < 1 \times 10^{-5}$; blue line) with hippocampal atrophy rates were identified.

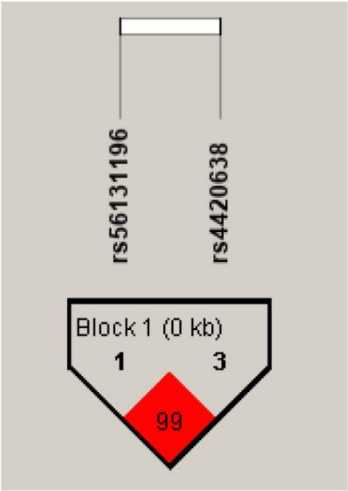

Supplementary Figure 3. Plot of linkage disequilibrium between rs4420638 and rs56131196. R^2=0.987, D'=0.993.

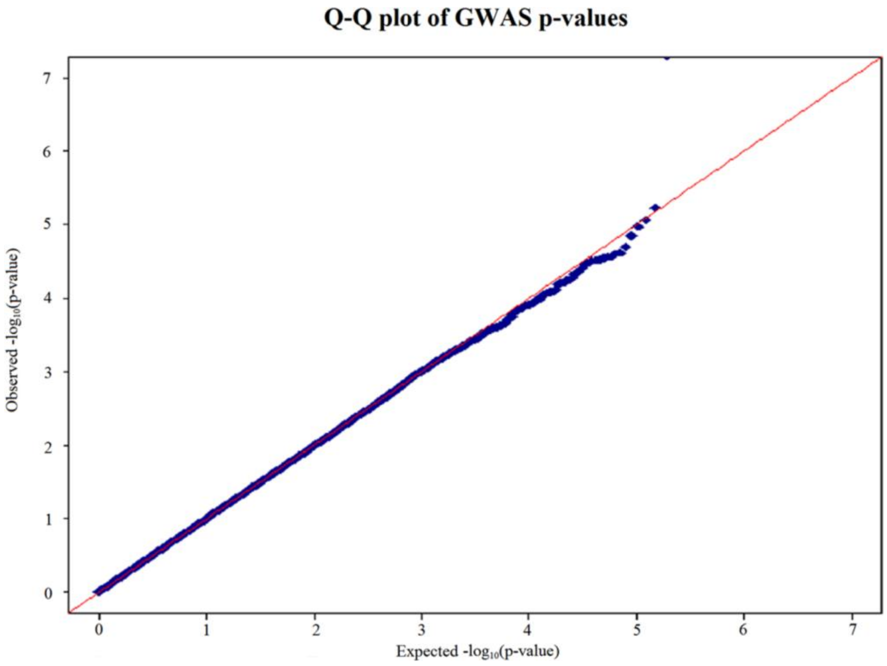

Supplementary Figure 4: Quantile-Quantile plot. Abbreviations: GWAS, genome-wide association study; Q-Q plot, Quantile-Quantile plot.

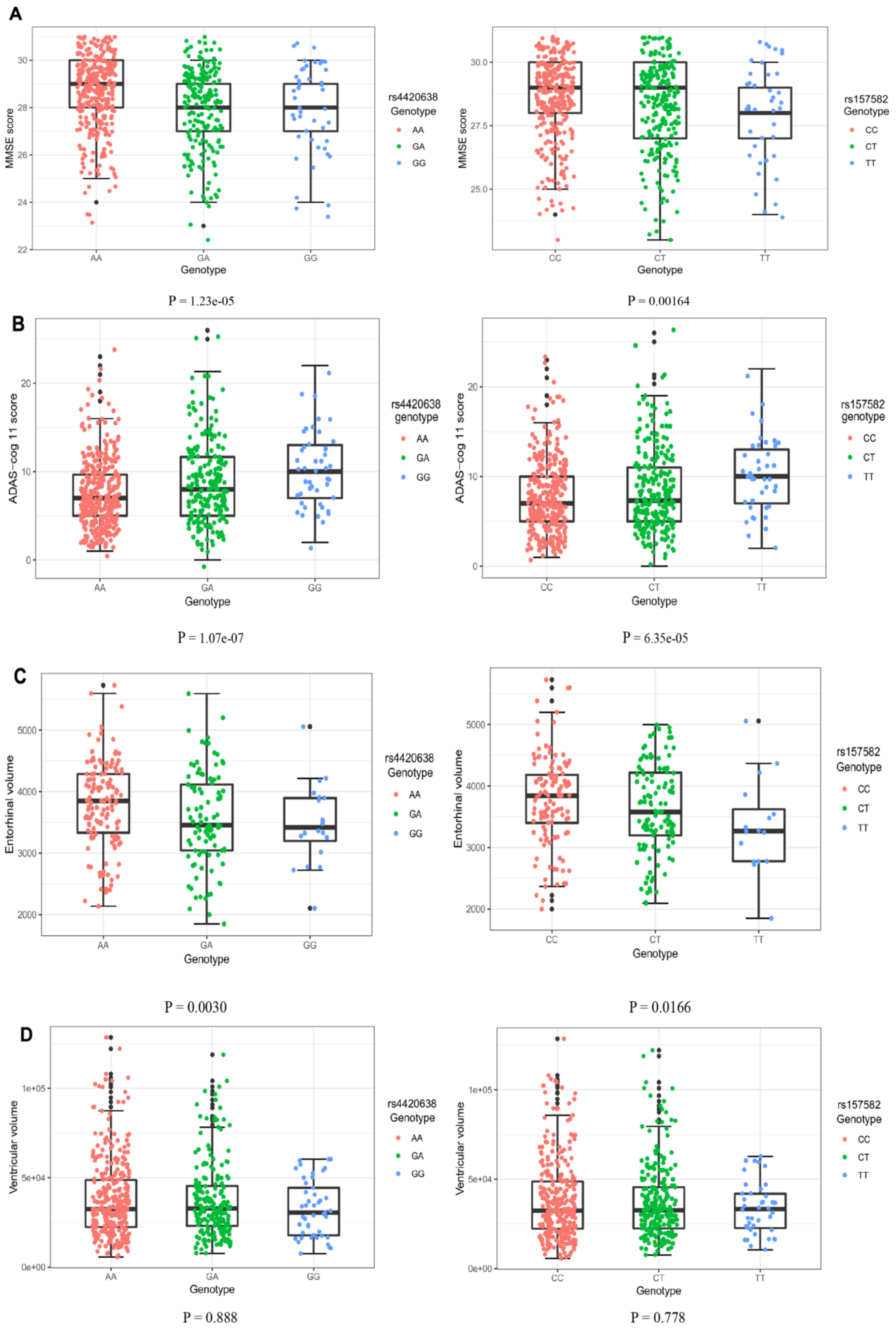

Supplementary Figure 5. Impact of rs4420638 and rs157582 on cognitive scores and brain structures at baseline. (**A**) Mini-mental State Examination (MMSE) score. (**B**) Alzheimer Disease Assessment Scale-cognitive subscale 11 (ADAS-cog 11) score. (**C**) Entorhinal volume. (**D**) Ventricular volume.

A

Rs4420638

Trans-eQTLs

P-value	SNP	SNP Chr.	SNP Chr. position	Probe	Probe Chr.	Probe Chr. position	SNP Alleles	Minor Allele	Z-score	Gene name	FDR
1.4287226161643386E-5	rs4420638	19	50114786	4070039	13	88664889	G/A	G	4.34	-	0.48

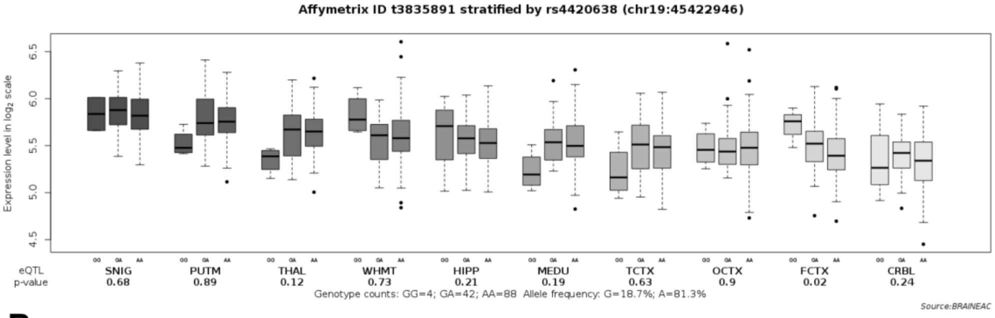

B

Rs56131196

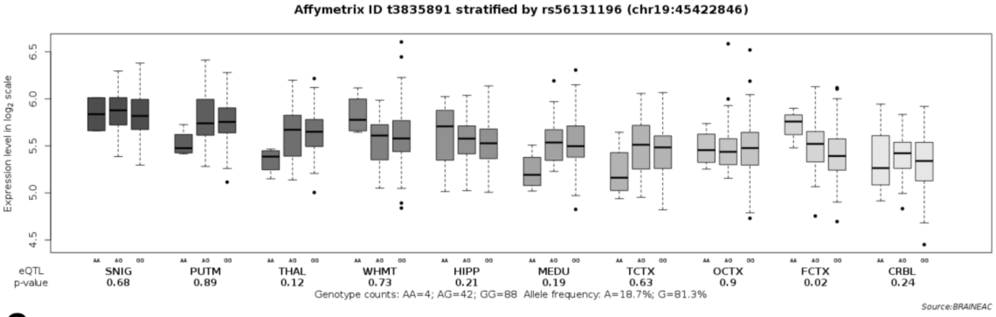

C

Rs157582

Cis-eQTLs

P-value	SNP	SNP Chr.	SNP Chr. Position	Probe	Probe Chr.	Probe Chr. position	SNP Alleles	Minor Allele	Z-score	Gene name	FDR
0.002408548933182936 7	rs157582	19	50088059	110605	19	50286067	T/C	T	3.03	GEMIN7	0.41

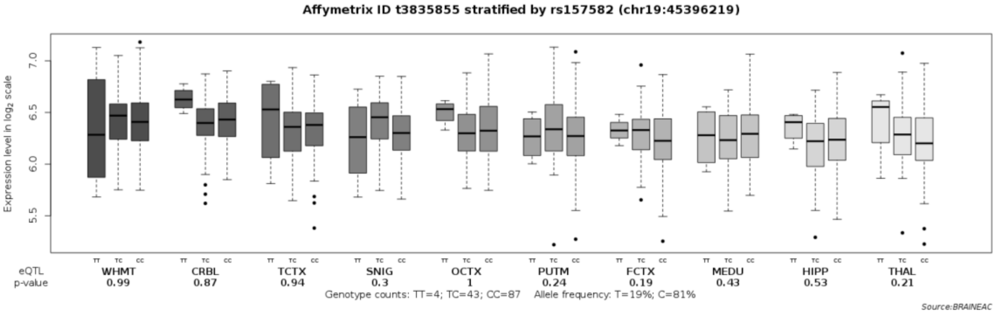

Supplementary Figure 6. Bioinformatics analyses. Abbreviations: Chr, chromosome; CRBL, cerebellar cortex; eQTL, expression quantitative trait loci; FCTX, frontal cortex; FDR, false discovery rate; HIPP, hippocampus; log, logarithm; MEDU, medulla; OCTX, occipital cortex; PUTM, putamen; SNIG, substantia nigra; SNP, single nucleotide polymorphism; TCTX, temporal cortex; THAL, thalamus; WHMT, intralobular white matter.
