# Supplementary material for: Genome-wide association study of hippocampal atrophy rate in non-demented elders
Source: Aging (Albany NY). 2019 Nov 23;11(22):10468–84. doi: 10.18632/aging.102470 (PMC6914394; doi:10.18632/aging.102470)
Supplement: Supplementary Tables [file aging-11-102470-s001.pdf]

SUPPLEMENTARY TABLES

Supplementary Table 1. Suggestive SNPs related to hippocampal atrophy rates without *APOE* ε4 fitted as a covariate.

CHR	BP	SNP	MAF	Closest Gene	SNP Type	BETA	P
19	45395619	rs2075650	G=0.1194	*TOMM40*	intron	−0.005827	5.11E-08
18	27797293	rs4271662	A=0.3606	*MIR302F*	intergenic	−0.003789	5.86E-06
18	27801582	rs2900721	T=0.3458	*MIR302F*	intergenic	−0.00372	8.76E-06

Abbreviations: BP, base pair (variant position); CHR, chromosome; MAF, minor allele frequency; SNP, single nucleotide polymorphism.

Supplementary Table 2. Top SNPs related to hippocampal atrophy rates with *APOE* ε4 fitted as a covariate.

CHR	BP	SNP	MAF	Closest Gene	SNP Type	BETA	P
18	27797293	rs4271662	A=0.3606	*MIR302F*	intergenic	-0.003464	1.68E-05
4	66628221	rs4382087	C=0.4427	*LOC100144602*	intergenic	0.003585	1.96E-05
15	100819272	rs7494886	C=0.1394	*ADAMTS17*	intron	-0.003804	1.97E-05
11	70002987	rs3781658	A=0.4655	*ANO1*	intron	-0.003635	2.20E-05
6	157915204	rs2365386	T=0.3984	*ZDHHC14*	intron	0.003528	2.27E-05
18	27801582	rs2900721	T=0.3458	*MIR302F*	intergenic	-0.003413	2.28E-05
12	69477770	rs12582988	A=0.1430	*CPM*	intergenic	0.004236	2.53E-05
10	81061724	rs10824740	A=0.3472	*ZMIZ1*	intron	-0.003841	2.62E-05
4	189850773	rs10001577	T=0.4493	*LOC285442*	intergenic	0.003894	2.74E-05
4	189860582	rs62341079	T=0.4171	*LOC285442*	intergenic	0.003852	3.08E-05
21	40024830	rs459813	T=0.2865	*ERG*	intron	0.004252	3.42E-05
3	61834942	rs6766943	G=0.3464	*PTPRG*	intron	0.004118	3.60E-05
4	96811611	rs4699474	G=0.2482	*PDHA2*	intergenic	-0.004146	3.62E-05
14	105149313	rs12884142	C=0.1208	*MIR4710*	intergenic	-0.003959	3.97E-05
4	189862164	rs12647666	T=0.4169	*LOC285442*	intergenic	0.003785	4.01E-05
6	19824025	rs6921758	T=0.4718	*LOC100506885*	intergenic	-0.003646	4.20E-05
3	151911863	rs323613	G=0.2316	*LOC101928166*	intron	-0.00401	4.46E-05
6	31069489	rs9263565	T=0.3165	*C6orf15*	intergenic	0.003405	4.47E-05
1	4626267	rs241272	G=0.4607	*AJAP1*	intron	0.003785	4.55E-05
2	139029250	rs6710702	T=0.2165	*RPL15P5*	intergenic	-0.003783	4.62E-05
19	2430232	rs743578	C=0.3878	*LMNB2*	intron	-0.00364	4.80E-05
6	112999076	rs4945909	G=0.4718	*PA2G4P5*	intergenic	0.003288	4.82E-05
15	100807344	rs11634485	T=0.3820	*ADAMTS17*	intron	-0.003412	4.93E-05
10	81064719	rs2296425	A=0.2648	*ZMIZ1*	intron	-0.004008	5.00E-05

Abbreviations: BP, base pair (variant position); CHR, chromosome; MAF, minor allele frequency; SNP, single nucleotide polymorphism.

Supplementary Table 3. COX regression with age and gender as covariates among different genotypes of rs4420638 and rs157582.

Rs4420638

Omnibus Tests of Model Coefficients							
Step	-2 LOG Likelihood	Overall (score)			Change From Previous Block		
		Chi-square	df	Sig.	Chi-square	df	Sig.
1	2077.519	47.256	2	0.000	41.495	2	0.000

a. Beginning Block Number 1. Method = Forward Stepwise (Likelihood Ratio)

Variables in the Equation								
	B	SE	Wald	df	Sig.	Exp (B)	95.0% CI for Exp (B)	
							Lower	Upper
Step 1 group			43.104	2	0.000			
group (1)	1.365	0.234	34.142	1.000	0.000	3.917	2.478	6.191
group (2)	0.794	0.160	24.504	1.000	0.000	2.212	1.615	3.029

Note: Group represents AA genotype; group (1) represents GG genotype; group (2) represents AG genotype.

Rs157582

Omnibus Tests of Model Coefficients							
Step	-2 LOG Likelihood	Overall (score)			Change From Previous Block		
		Chi-square	df	Sig.	Chi-square	df	Sig.
1	1715.267	34.083	2	0.000	26.558	2	0.000

a. Beginning Block Number 1. Method = Forward Stepwise (Likelihood Ratio)

Variables in the Equation								
	B	SE	Wald	df	Sig.	Exp (B)	95.0% CI for Exp (B)	
							Lower	Upper
Step 1 group			30.620	2	0.000			
group(1)	1.433	0.263	29.672	1.000	0.000	4.190	2.502	7.016
group(2)	0.533	0.179	8.911	1.000	0.003	1.704	1.201	2.418

Note: Group represents CC genotype; group (1) represents TT genotype; group (2) represents CT genotype.
Abbreviations: B, beta; CI, confidence interval; df, degrees of freedom; Exp, exponent; LOG, logarithm; SE, standard error; Sig, significance.
